# A Comprehensive Review of Splenic Cysts: Case Reports and Clinical Insight

**DOI:** 10.7759/cureus.73502

**Published:** 2024-11-12

**Authors:** Ibrahim I Ibikunle, Sekinat O Olaboopo, Suma Kaza

**Affiliations:** 1 Pathology, Avalon University School of Medicine, Willemstad, CUW

**Keywords:** accurate diagnosis, primary and secondary splenic cysts, pseudocysts, splenic cysts, surgical techniques

## Abstract

Splenic cysts, although rare, present a diagnostic and therapeutic challenge due to their varied etiologies and clinical manifestations. This review synthesizes the current understanding of splenic cysts through an analysis of thirteen case reports spanning from 1988 to 2023. Etiologies include epithelial, parasitic, and sarcomatous origins. Diagnostic modalities, including imaging and histopathology, facilitate accurate classification and inform subsequent management decisions. Various surgical techniques, including laparoscopic and spleen-preserving approaches, have been employed with favorable outcomes. This review provides insights into the pathogenesis, classification, diagnostic workup, and treatment modalities of splenic cysts based on collective experiences reported in the literature.

## Introduction and background

Splenic cysts are relatively rare clinical entities, with an incidence reported in autopsies ranging from 0.07% to 0.13%. They are classified into parasitic and non-parasitic cysts, with the latter further divided into primary (true) cysts and secondary (false or pseudocysts). Understanding the underlying pathogenesis, diagnostic strategies, and therapeutic approaches is crucial for optimal management. This review aims to explore the spectrum of non-parasitic splenic cysts through an analysis of selected case reports, providing insights into their epidemiology, pathogenesis, clinical manifestations, diagnostic methodologies, and management strategies by consolidating existing knowledge on splenic cysts through an analysis of thirteen case reports spanning over three decades.

Search strategy

This review synthesizes data from 13 case reports (2003-2023) to analyze splenic cyst characteristics, diagnostics, and treatment strategies across diverse patient demographics. To ensure a comprehensive dataset, case reports were searched using PubMed, MEDLINE, and EMBASE. We combined search terms including "splenic cyst", "non-parasitic", "epithelial cyst", and "pseudocyst". The cases included represent a range of patient demographics, encompassing both pediatric and adult patients, with ages ranging from 10 to 50 years and including both male and female patients with various presentations and treatments. This allows for insights into how age, gender, and cyst type influence management.

Diagnostic methods primarily included ultrasound, CT, and MRI, with fine-needle aspiration cytology and biopsies utilized in cases with suspected malignancy. Therapeutic approaches ranged from conservative observation for small, asymptomatic cysts to laparoscopic cystectomy and spleen-preserving surgeries for larger cysts, particularly in younger patients. More aggressive interventions, such as partial or total splenectomy, were reserved for cases with malignancy risk or recurrence. Data extraction was conducted independently by two reviewers to minimize bias and ensure consistency in interpretation.

Classification and pathogenesis

**Figure 1:**
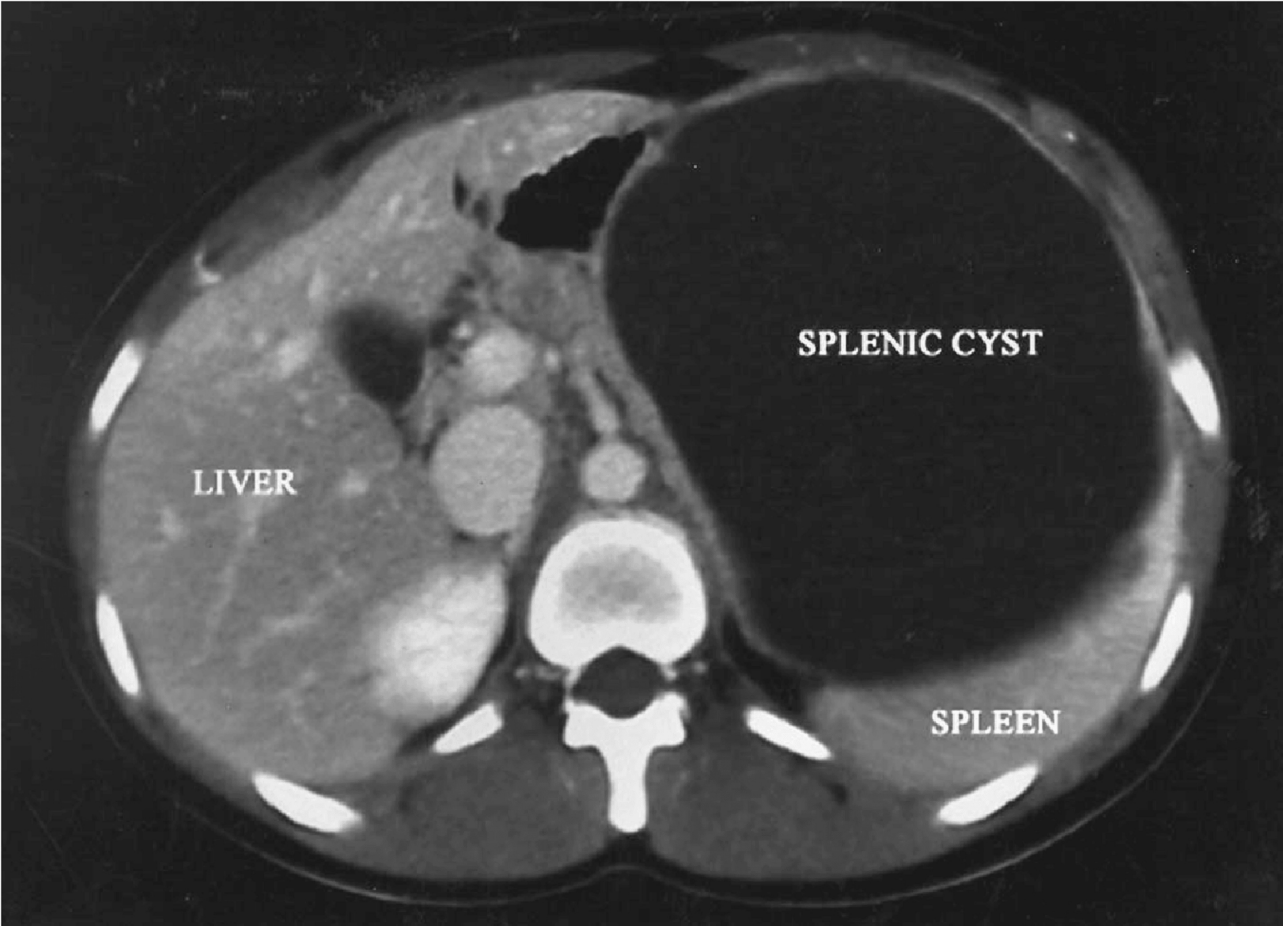
Computed tomography of the abdomen showing a 16 cm diameter fluid-filled epithelial splenic cyst in the spleen. Used with permission from Palmieri et al. [3]

**Figure 2:**
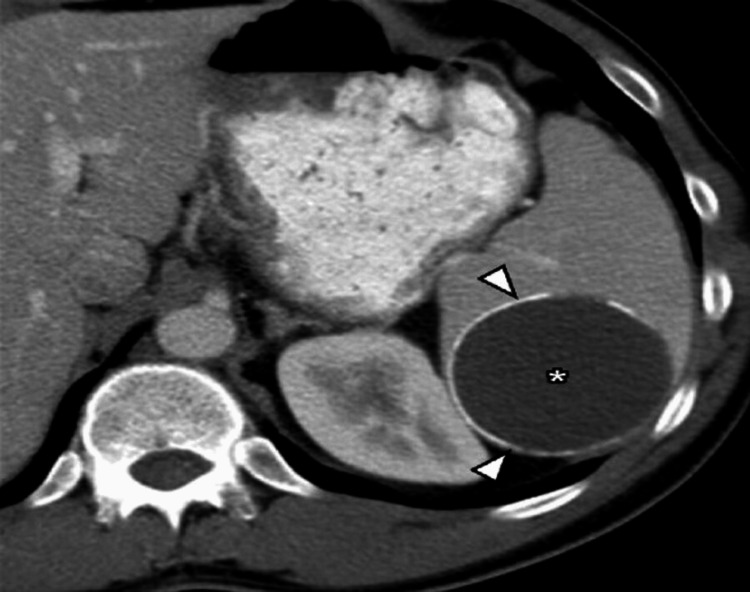
Splenic pseudocyst. Contrast-enhanced CT shows a well-defined cyst in the spleen (*) with a peripheral rim of calcification (arrowheads) and no internal septations, presumed to be a post-traumatic pseudocyst. Used with permission from Kaza et al. [4]

Non-parasitic splenic cysts can be classified based on their etiology into true cysts (Figure 1), which have an epithelial lining, and pseudocysts (Figure 2), which do not. True cysts are often congenital, whereas pseudocysts typically result from trauma or infarction. The pathogenesis of non-parasitic splenic cysts varies depending on the type. True cysts may arise from developmental anomalies in the splenic tissue, while pseudocysts are generally caused by trauma or hematologic disturbances. Epithelial cysts, accounting for the majority of cases, originate from the splenic parenchyma or mesothelial lining. Studies by Bürrig [1] highlight the mesothelial origin of some true cysts, providing cellular insights into their development. Parasitic cysts, such as hydatid cysts, result from *Echinococcus granulosus* infestation. Sarcomatous cysts, although rare, present diagnostic challenges due to their malignant potential. Studies by Bürrig and Morgenstern offer detailed classifications and discuss the pathogenesis related to these distinctions [1,2]. Table 1 presents a concise classification and characterization of different types of splenic cysts.

**Table 1:** Classification and characteristics of splenic cysts [5]

Type of Splenic Cyst	Etiology	Pathogenesis	Key Characteristics	Common Treatments
True cysts (primary)	Congenital	Developmental anomalies in the splenic tissue; mesothelial origin	Epithelial lining, typically solitary, may grow large	Cyst excision, partial splenectomy
Pseudocysts (secondary)	Trauma, hematologic disturbances	Result from trauma or infarction	Lack epithelial lining, often irregular shape	Observation, cyst marsupialization
Parasitic cysts	Echinococcus granulosus infestation	Parasitic infection	Often multiple, can cause hydatid disease	Antiparasitic treatment, cyst removal
Sarcomatous cysts	Rare, malignant potential	Neoplastic origin	May mimic benign cysts but have malignancy risk	Splenectomy, oncologic follow-up

Diagnostic approaches and management strategies

The diagnosis of splenic cysts relies on a combination of clinical evaluation, imaging modalities, and histopathological analysis. Patients with splenic cysts often present with non-specific symptoms such as abdominal pain, discomfort, and bloating. In some cases, as detailed by Amr et al. [6] and Ashmore et al. [7], cysts may remain asymptomatic and are discovered incidentally during imaging for unrelated conditions.

Diagnostic imaging is crucial for identifying and classifying splenic cysts. Ultrasound, CT scans, and MRI play pivotal roles, as evidenced in cases reported by Avital and Kashtan [8] and Daga et al. [9]. These modalities help determine the size, location, and nature (simple vs. complex, cystic vs. solid) of the cysts, which is essential for planning management. Fine-needle aspiration cytology (FNAC) or core needle biopsy may be performed to confirm the diagnosis and rule out malignancy.

**Figure 3:**
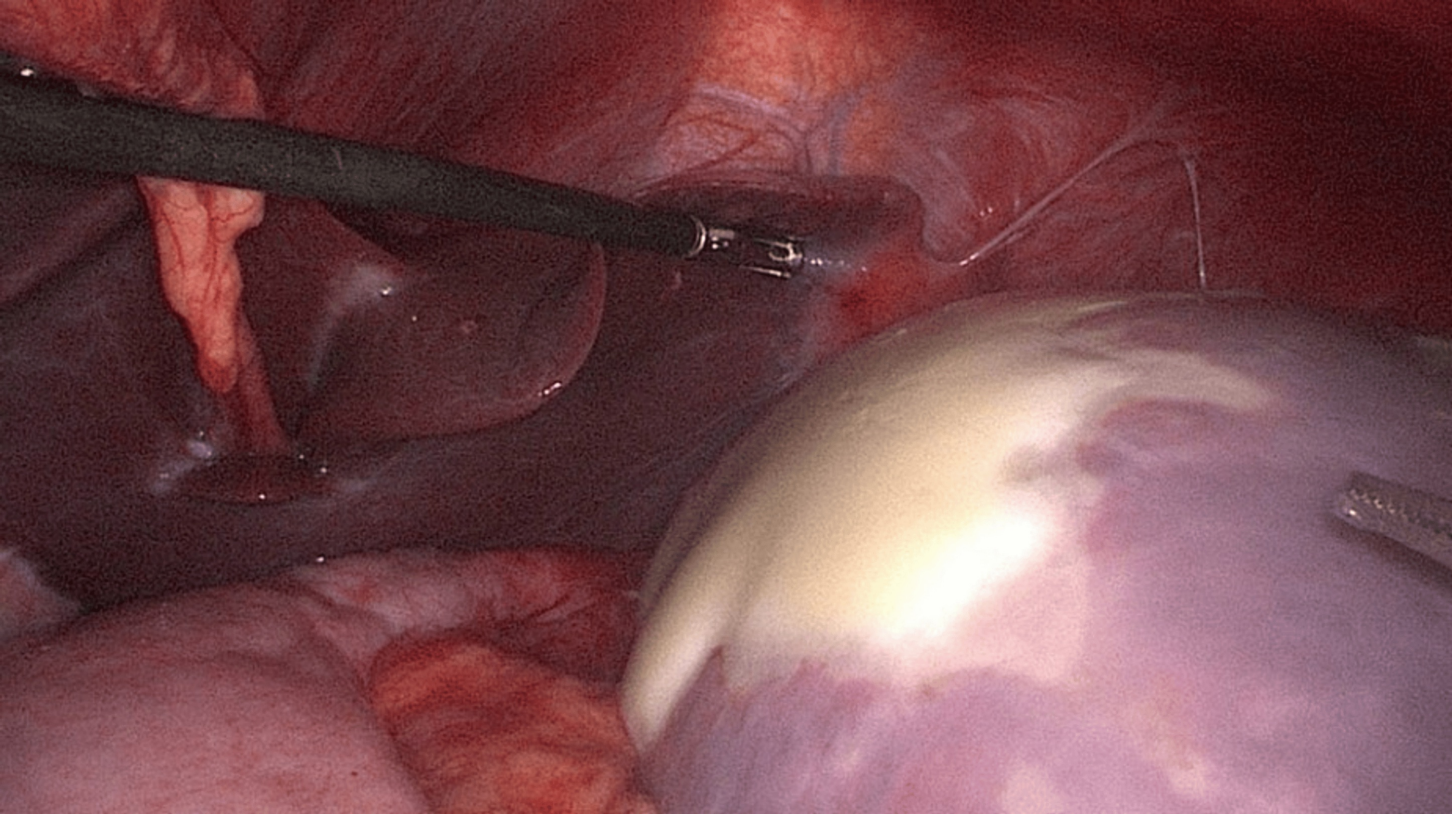
Intraoperative photograph showing diagnostic laparoscopy demonstrating a splenic cyst. Used with permission from Kumar et al. [12]

The management of splenic cysts ranges from conservative observation for asymptomatic, small cysts to surgical intervention for larger, symptomatic ones. Techniques such as laparoscopic cystectomy (Figure 3) and spleen-preserving surgeries are highlighted in reports by Zografos et al. and Geraghty et al., emphasizing advancements in minimally invasive surgery that offer reduced morbidity and preservation of splenic function [10,11].

## Review

Review of literature

Each of the cases selected for this review illustrates unique aspects of splenic cysts along with their diagnostic challenges. For instance, Ashmore and Dasgupta [7] encountered a histologically confirmed splenic sarcoma initially masquerading as a cyst, underscoring the importance of thorough histopathological evaluation in guiding treatment decisions and prognostication. The complexity of diagnosing and managing splenic cysts is further highlighted in the case by Aloqaily et al. [13], where a large epidermoid cyst was initially misdiagnosed as a hydatid cyst. This emphasizes the critical role of differential diagnosis in splenic lesions and underscores the importance of serological and imaging studies in avoiding misdiagnosis. Similarly, Karbasian et al. [14] reported a giant non-parasitic splenic cyst, showcasing a case where imaging alone was insufficient for definitive diagnosis, necessitating histological examination to rule out malignancy. This influenced the surgical approach, ensuring complete removal while avoiding unnecessary total splenectomy.

Moreover, Kundal et al. [15] discuss the management of a giant epithelial cyst, while Kumar et al. [12] focus on spleen-preserving therapy in non-parasitic cysts. These reports collectively underscore the variability in clinical decisions based on cyst characteristics.

Another insightful case by Alharbi et al. [16] describes a large splenic cyst in a pediatric patient, presenting a unique challenge due to the patient's age and the potential implications for growth and immunological development. This report underscores the importance of conservative management strategies and regular monitoring in pediatric cases, where preserving splenic function is particularly crucial. The decision for surgical intervention, as noted in the case, was driven by symptomatic relief and the prevention of potential complications such as cyst rupture or infection, which are more consequential in pediatric patients. Table 2 provides an abridged summary of the case reports included in this analysis, highlighting key points such as cyst type, size, diagnostic methods, etc.

**Table 2:** Summary of case reports.

Reference	Patient Demographics	Cyst Type	Size of Cyst	Symptoms	Diagnostic Methods	Treatment	Outcome
Amr (2009) [6]	22-year-old female	Non-parasitic cyst	15 cm	Abdominal pain	Ultrasound, MRI	Total cyst excision	Symptom resolution
Ashmore et al. (2020) [7]	45-year-old female	Misdiagnosed sarcomatous cyst	12 cm	Abdominal pain	Ultrasound, MRI	Splenectomy	Symptom resolution
Avital and Kashtan (2003) [8]	35-year-old male	Epithelial cyst	10 cm	None	CT scan	Observation	No progression
Daga et al. (2011) [9]	12-year-old male	Epithelial cyst	12 cm	Abdominal pain	Ultrasound	Cyst excision	Symptom resolution
Zografos et al. (2010) [10]	30-year-old male	Pseudocyst	8 cm	Abdominal pain	CT scan	Laparoscopic partial cystectomy	No recurrence
Geraghty et al. (2009) [11]	40-year-old female	True cyst	7 cm	Fullness, pain	Ultrasound, MRI	Laparoscopic excision	Full recovery
Aloqaily et al. (2023) [13]	32-year-old male	Epidermoid cyst (initially misdiagnosed as hydatid)	14 cm	Pain, mass effect	CT scan, serology	Cyst excision	No recurrence
Karbasian et al. (2023) [14]	28-year-old female	Giant non-parasitic cyst	22 cm	Abdominal pain, distension	CT scan, MRI	Cyst excision	Good recovery
Kundal et al. (2013) [15]	25-year-old female	Giant epithelial cyst	18 cm	Pain, palpable mass	Ultrasound, MRI	Cyst excision	Successful
Kumar et al. (2021) [12]	50-year-old male, 45-year-old female	Non-parasitic cysts	6-10 cm	Pain, fullness	CT scan	Spleen-preserving therapy	No complications
Alharbi et al. (2023) [16]	10-year-old female	Large cyst	20 cm	Abdominal distension	Ultrasound, CT scan	Cyst excision	Good recovery
Ingle et al. (2013) [17]	35-year-old male	Primary epithelial cyst	10 cm	Abdominal discomfort	Ultrasound, CT scan	Cyst excision	Full recovery
Macheras et al. (2005) [18]	Various ages, both genders	Non-parasitic true cysts	5-12 cm	Pain, mass effect	Ultrasound, CT scan	Partial splenectomy	Successful, no recurrence

Discussion

The management of non-parasitic splenic cysts remains a nuanced and evolving field. The decision to intervene surgically is often influenced by factors such as cyst size, the presence of symptoms, and potential complications, including rupture or infection. For example, large cysts frequently necessitate surgical intervention to prevent complications and provide symptomatic relief. However, the choice between total splenectomy and spleen-preserving techniques, such as cystectomy or partial splenectomy, depends on multiple considerations, including the patient's age, cyst location, and the presence of healthy splenic tissue. The increasing preference for spleen-preserving surgeries is driven by their potential to reduce postoperative complications and preserve immune function.

Advancements in minimally invasive techniques, particularly laparoscopic and robotic-assisted surgeries, have revolutionized the management of splenic cysts. These approaches offer benefits such as reduced postoperative pain, shorter hospital stays, and faster recovery times compared to traditional open surgery. Numerous studies have demonstrated the efficacy of laparoscopic techniques in managing large splenic cysts, highlighting their safety and feasibility. Additionally, robotic-assisted surgery, with its enhanced precision and dexterity, holds promise for complex cases requiring meticulous dissection and preservation of splenic tissue. Despite these advancements, the need for rigorous clinical trials and long-term follow-up studies to validate the superiority of minimally invasive techniques over conventional methods remains evident.

The complexity of managing pediatric splenic cysts is another critical aspect discussed in this review. Surgical intervention in pediatric patients carries implications beyond immediate clinical outcomes, potentially impacting growth and immune development. Conservative management, involving regular monitoring and delayed surgical intervention, is often recommended for asymptomatic cases to avoid surgical risks. However, symptomatic cysts or those with complications necessitate timely surgical intervention. This delicate balance between conservative and surgical management underscores the need for standardized guidelines tailored to pediatric patients, with long-term follow-up to assess growth and immunological outcomes.

The variability in clinical presentations and management strategies for splenic cysts calls for a more systematic approach to diagnosis and treatment. Imaging modalities, including ultrasound, CT, and MRI, play a pivotal role in the initial evaluation and characterization of cysts. However, as demonstrated in multiple cases, imaging alone may sometimes be insufficient for a definitive diagnosis, necessitating histopathological examination to differentiate between benign and malignant lesions. This underscores the critical importance of a multidisciplinary approach, involving radiologists, pathologists, and surgeons, in the comprehensive management of splenic cysts. Furthermore, the integration of advanced imaging techniques and molecular diagnostics could enhance the accuracy of preoperative diagnosis, guiding more precise and individualized treatment plans.

In conclusion, the management of non-parasitic splenic cysts necessitates a multifaceted approach, taking into account cyst characteristics, patient demographics, and available surgical expertise. While advancements in minimally invasive techniques offer promise for improved outcomes, the importance of individualized patient care and the development of standardized guidelines cannot be overstated. Future research should focus on large-scale, multicenter studies to establish evidence-based protocols that optimize patient outcomes, with an emphasis on organ preservation and quality of life.

## Conclusions

Splenic cysts represent a heterogeneous group of lesions with varied etiologies and clinical presentations. A systematic diagnostic approach, including imaging and histopathological analysis, is essential for accurate classification and subsequent management decisions. Surgical intervention remains the cornerstone of treatment, with an increasing emphasis on spleen-preserving techniques. Further research is warranted to optimize management strategies and improve long-term outcomes for splenic cysts.
